# An Insight into the Proteome of *Crithidia fasciculata* Choanomastigotes as a Comparative Approach to Axenic Growth, Peanut Lectin Agglutination and Differentiation of *Leishmania* spp. Promastigotes

**DOI:** 10.1371/journal.pone.0113837

**Published:** 2014-12-11

**Authors:** Pedro J. Alcolea, Ana Alonso, Francisco García-Tabares, Alfredo Toraño, Vicente Larraga

**Affiliations:** 1 Department of Molecular Microbiology and Biology of Infections and Service of Proteomics and Genomics, Centro de Investigaciones Biológicas (Consejo Superior de Investigaciones Científicas), Madrid, Spain; 2 Servicio de Inmunología, Centro Nacional de Microbiología, Virología e Inmunología Sanitarias (Instituto de Salud Carlos III), Majadahonda, Madrid, Spain; Instituto Oswaldo Cruz, Fiocruz, Brazil

## Abstract

The life cycle of the trypanosomatid *Crithidia fasciculata* is monogenetic, as the unique hosts of these parasites are different species of culicids. The comparison of these non-pathogenic microorganisms evolutionary close to other species of trypanosomatids that develop digenetic life cycles and cause chronic severe sickness to millions of people worldwide is of outstanding interest. A ground-breaking analysis of differential protein abundance in *Crithidia fasciculata* is reported herein. The comparison of the outcome with previous gene expression profiling studies developed in the related human pathogens of the genus *Leishmania* has revealed substantial differences between the motile stages of these closely related organisms in abundance of proteins involved in catabolism, redox homeostasis, intracellular signalling, and gene expression regulation. As *L. major* and *L. infantum* agglutinate with peanut lectin and non-agglutinating parasites are more infective, the agglutination properties were evaluated in *C. fasciculata*. The result is that choanomastigotes are able to agglutinate with peanut lectin and a non-agglutinating subpopulation can be also isolated. As a difference with *L. infantum*, the non-agglutinating subpopulation over-expresses the whole machinery for maintenance of redox homeostasis and the translation factors eIF5a, EF1α and EF2, what suggests a relationship between the lack of agglutination and a differentiation process.

## Introduction

Protists of the genus *Crithidia* (Léger, 1902) (Kinetoplastida: Trypanosomatidae) are flagellate parasites that exclusively infect insects [1]. The numerous species of *Crithidia* have broad host specificity and are able to parasitize a variety of species grouped into the orders Diptera, Hemiptera and Himenoptera. Specificity also varies importantly depending on the species of the parasite [1]. Particularly, *C. fasciculata* successfully infects many species of mosquitoes.

**Figure 1:**
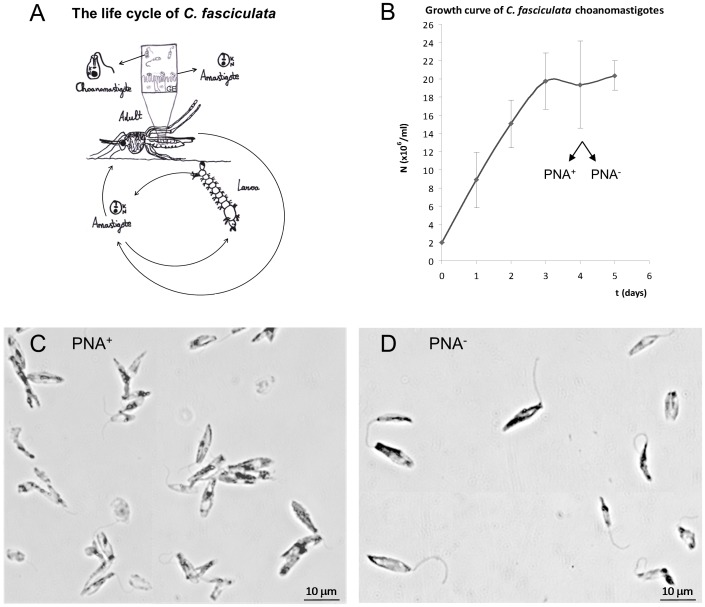
Life cycle, growth kinetics and peanut lectin agglutination of *C. fasciculata* choanomastigotes. (A) The monogenetic life cycle of *C. fasciculata* involves a culicid host, where amastigotes attached to the gut epithelium and voided in faeces are disseminated in the environment and orally passed to other hosts at any of the developmental stages. Choanomastigotes are the motile stage that allows the colonization of the gut of the host. GE: gut epithelium. Adapted from Olsen, 1974. (B) Average growth curve of three *C. fasciculata* choanomastigote cultures (three biological replicates). Total proteins were extracted every day until the culture reached the stationary phase. N is the average cell density. (C) and (D) 10% Giemsa staining of the PNA^+^ and PNA^-^ *C. fasciculata* choanomastigote subpopulations within the stationary phase of axenic culture, respectively.

Although these parasites are polymorphic, two stages are clearly distinguished. Choanomastigotes are free-swimming stumpy cells characteristic of this genus that are round in their posterior part and truncated in the apical pole by the funnel-shaped flagellar pocket close to the kinetoplast, which is slightly anterior to the nucleus. Amastigotes are non-motile round cells with a flagellum non-emergent from the cellular body. Therefore, they are morphologically similar to amastigotes of the genus *Leishmania*, although they are extracellular (reviewed in [2]). The life cycle of *C. fasciculata* is developed in the gut of the culicid, which becomes infected by ingestion of amastigotes voided with feces of other hosts. Then, amastigotes undergo a differentiation process into choanomastigotes to ensure proper colonization of the gut. Choanomastigotes differentiate back into non-motile round amastigotes that are attached to the gut epithelium by hemidesmosomes [3] frequently leading to damage [4]. Infected adult mosquitoes contaminate aquatic environments with amastigotes as well as flowers when they feed on nectar, thus providing chances for transmission of the parasite. Amastigotes are released within the feces or the entire body of the dead insect. Eventually, the larval and pupal instars of mosquitoes get infected in the aquatic habitat and finally amastigotes are transmitted to the adult mosquito through the metamorphosing gut [2] leading to completion of the life cycle (Fig. 1A).

Parasites grouped into the genus *Crithidia* develop monogenetic life cycles involving the extracellular choanomastigote and amastigote stages, and consequently do not infect mammals. The comparison with species of the same family developing digenetic life cycles responsible for leishmaniasis and trypanosomiasis is of outstanding interest. Even though these parasites afflict millions of people worldwide, they are still neglected [5]. As a difference with *Leishmania* spp., parasites from the genus *Crithidia* are not pathogenic to humans. For this reason, their biology at the molecular and cellular levels remains almost unexplored despite their evolutionary relation with the genus *Leishmania* (reviewed in [6]). Advantageously, both organisms are closely related at the crown of the phylogenetic tree of trypanosomatids [6], [7], [8], [9] despite their different life cycles.

There has been no attempt to quantify differential transcript and protein abundance at medium or large scale in any of the *Crithidia* species so far. The comparison between monogenetic and digenetic trypanosomatids may contribute to explain the mechanisms of adaptation to different hosts in the latter, which are mammals in the case of *Leishmania* spp. and *Trypanosoma* spp. This study is, to our knowledge, the first insight into the proteome of *C. fasciculata* choanomastigotes in axenic culture and has been performed by two dimension electrophoresis (2DE)-based analysis and protein identification by MALDI-TOF/TOF tandem mass spectrometry. The recent release of the *C. fasciculata* genome sequence and annotations has led to successful identification of most of the spots analyzed. Agglutination of choanomastigotes with PNA has been tested with positive results and a proteome analysis of the PNA^+^ and PNA^-^ subpopulations in stationary phase has also been performed. The PNA^-^ subpopulation is more infective in *L. major* [10] and *L. infantum* [11] but the implications of the existence of this subpopulation in *Crithidia* spp. revealed herein is more likely related to development only. The new proteomic data, including the PNA^+^ and PNA^-^ subpopulations, have been compared with the outcome of published stage-specific transcriptome and proteome analyses in the genus *Leishmania*, what has revealed differences in abundance of proteins involved in gene expression regulation, carbohydrate metabolism, redox homeostasis and other processes.

## Materials and Methods

### Parasite cultures

Choanomastigotes of the *C. fasciculata* strain LLM494 [12] were cultured at 27°C in complete medium containing RPMI 1640 medium supplemented with L-glutamine (Life Technologies, Carlsbad, CA), 10% heat inactivated foetal bovine serum (Lonza, Basel, Switzerland) and 100 µg/ml streptomycin – 100 IU/ml penicillin (Life Technologies) pH 7.2. Cell density of three replicate cultures started at 2×10^6^ cells/ml was monitored and choanomastigotes were harvested daily at 2,000 g for 10 min and washed once with PBS at 4°C.

### Purification of PNA

100 g of non-roasted peanuts were submerged in 300 ml PBS at 4°C overnight, mashed and filtered through a gauze squeezing the homogenate to collect as much liquid as possible. Then, the homogenate was centrifuged at 8000 rpm for 10 min in a Sorvall RC5C centrifuge using a GSA rotor (Dupont, Stevenage, Herts, UK). The supernatant was recovered and clarified through filter paper. Then, 40% (w/v) (NH_4_)SO_4_ was progressively dissolved in the extract, which was then incubated at room temperature for 30 min. After 20 min of centrifugation at 6000 rpm, the supernatant was recovered and (NH_4_)SO_4_ progressively dissolved up to 75% (w/v). The extract was centrifuged again and the pellet recovered, resuspended in 50 ml PBS and dialyzed three times with 40 volumes of PBS at 4°C overnight. To clarify the extract, an additional centrifugation step at 6000 rpm for 10 min using an SS34 rotor and three filtration steps were carried out, the first one through filter paper, the second one through a 47 mm diameter, 2 µm pore size borosilicate fiber pre-filter (Millipore, Billerica, MA) and the third one through BioGel A2 (BioRad, Hercules, CA). Finally, the PNA was purified by affinity chromatography using melibiose immobilized in agarose beads (Sigma Aldrich, Buchs, Switzerland). Two washes with PBS were performed and the lectin eluted with 50 mM D-galactose (Sigma). The 1 ml fractions collected were quantified by the Warburg and Christian method [13].

### Separation of PNA^+^ and PNA^-^ stationary phase choanomastigote subpopulations

Stationary phase choanomastigotes were resuspended at 2 x 10^8^ cells/ml in complete medium containing 50 µg/ml PNA in a polypropylene tube. After 30 min incubation at room temperature allowing the sedimentation of agglutination complexes, the supernatant was recovered and the sediment was resuspended again in complete medium containing PNA at the same concentration. Sedimentation of both fractions was performed again but this time at 200 g for 10 min. The resulting supernatants were mixed and centrifuged at 2,000 g for 10 min to harvest PNA^-^ promastigotes, whereas only the pellet obtained from the original sediment was processed as the PNA^+^ fraction. All the steps of this procedure were checked at the light microscope (63X).

### Preparation and quantification of protein extracts

We described a similar proteome analysis procedure [14] and modifications are detailed herein. Each sample of parasites was resuspended in 300 µl lysis buffer (8.4 M urea, 2.4 M thiourea, 5% CHAPS, 50 mM DTT, 1% Triton X-100, 50 µg/ml DNase and Mini EDTA-free Protease Inhibitor Cocktail according to the manufacturer's instructions –Roche, Mannheim, Germany). The total protein extracts obtained were agitated by mild rotation at 4°C for 30 min, centrifuged at 8,000 g for 10 min and precipitated with methanol/chloroform [15]. All samples were dried at room temperature for 5 min and resuspended in 2X rehydration buffer (7 M urea, 2 M thiourea, 4% CHAPS, 0.003% bromophenol blue). Protein quantification was performed by the *RC DC protein assay* kit (BioRad). 50 µg aliquots of each sample were diluted to a final volume of 140 µl in 2X buffer containing 18.2 M DTT and 0.5% IPG buffer solution pH 3–10 (BioRad). Further confirmation was carried out by densitometric analysis of 10% PAGE-SDS gels [16] as described [14].

### 2DE separation and analysis of protein abundance

Isoelectrofocusing of 50 µg total protein per sample was performed on IPG strips (non-linear pH 3–10 gradient, 7 cm, BioRad) in a *Protean IEF Cell* system (BioRad) following the manufacturer's instructions. A seven step program was used (50 V for 12 h, 250 V for 1 h, 500 V for 1 h, 1000 V for 1 h, 2000 V for 1 h, linear ramp to 8000 V for 1 h and 8000 V up to 3500 V·h). More than a total of 12,000 V·h were reached in all runs. The second dimension was run by 12% SDS-PAGE in a pre-cooled *MiniProtean 3 Dodeca Cell* system (BioRad) at 0.5 W/gel for 30 min and then at 1.5 W up to 5 min after the die-front reached the bottom edge of the gels (approximately 2 h). Then, the gels were stained with SYPRO Ruby protein gel stain (BioRad) following the manufacturer's instructions. Imaging was performed with *EXQuest Spot Cutter* system and the analysis of differential abundance with *PDQuest 2D Advanced 8.0.1* software (BioRad) following the manufacturer's instructions. First, all spots were automatically detected and thereafter manually checked by observation of single spot quantitation histograms and 2DE gel images. Normalized intensities were calculated by the *Total Quantity in Valid Spots* algorithm to ensure that relative quantification between gels is not biased by staining and background. The statistical analysis was performed by the Student's t-test at 0.05 significance level. Three replicates of each experiment were performed.

### Protein identification by MALDI-TOF/TOF mass spectrometry

The spots selected in the previous analysis were excised with *EXQuest Spot Cutter* (BioRad), digested with trypsin and prepared for MALDI-TOF/TOF mass-spectrometry as we described [14]. A 0.8 µl drop of resuspended peptides from each spot was deposited in an *OptiTOF Plate* (Life Technologies) together with a 0.8 µl drop of a 3 g/l α-cyano-4-hydroxycinnamic solution (Sigma). The mixture was allowed to dry at room temperature. Samples were run in an *ABI 4800 MALDI-TOF/TOF* (Life Technologies) mass spectrometer in positive reflector mode at 25 kV for MS and 1 kV for MS/MS. The spectra were prepared with *ABI 4000 Series Explorer Software 3.6* (Life Technologies). Fingerprint and fragmentation spectra were run in *MASCOT 2.1* with *Global Protein Server Explorer 4.9* (Life Technologies) for protein identification with both the *NCBInr* database and annotations on the genome sequence of *C. fasciculata*. The genome sequence was completed at Washington University School of Medicine in St. Louis (Stephen Beverley, Richard Wilson) and assembly and annotations at Seattle Biomedical Research Institute (Peter Myler). These data can be retrieved from http://tritrypdb.org/common/downloads/release-8.0/CfasciculataCfCl/fasta/data/. A MIAPE-compliant report and the MS data have been deposited to the ProteomeXchange Consortium [17] via the PRIDE partner repository with the dataset identifier PXD001331 and DOI 10.6019/PXD001331.

### Western blot

Protein extracts were separated by SDS-PAGE in 8% slab gels (12 mA, 30 min; 30 mA, 90 min) in a *MiniProtean II Cell* system (BioRad). 20 µg protein extract was loaded per well including 1 µl Benzonase Nuclease HC (Novagen, Madison, WI). Blotting onto 0.45 µm nitrocellulose membranes (BioRad) was performed at 100 V for 1 h in a Mini Trans-Blot Cell wet transfer system (BioRad). Membrane blocking was carried out with 5% skimmed milk in PBS-0.1% Tween 20 (Sigma) for 1 h and washed three times with PBS-1% Tween 20 for 15, 5 and 5 min respectively. Next, membranes were incubated with 1∶500 of rabbit anti-LACK polyclonal serum for 2 h [18] or 1∶10,000 of monoclonal mouse anti-*L. mexicana* glycosomal GAPDH antibody kindly provided by Paul Michels (University of Edinburg) [19], washed again and incubated with 1∶2,000 HRP-conjugated goat anti-rabbit IgG (DAKO, Ely, UK) for 90 min. Once the wash steps were repeated, the immunoblots were developed using the ECL detection system (GE Healthcare, Pittsburg, PA) according to the manufacturer's instructions.

## Results and Discussion

### Growth kinetics of *C. fasciculata* choanomastigotes and 2DE-MS/MS analysis

**Figure 2:**
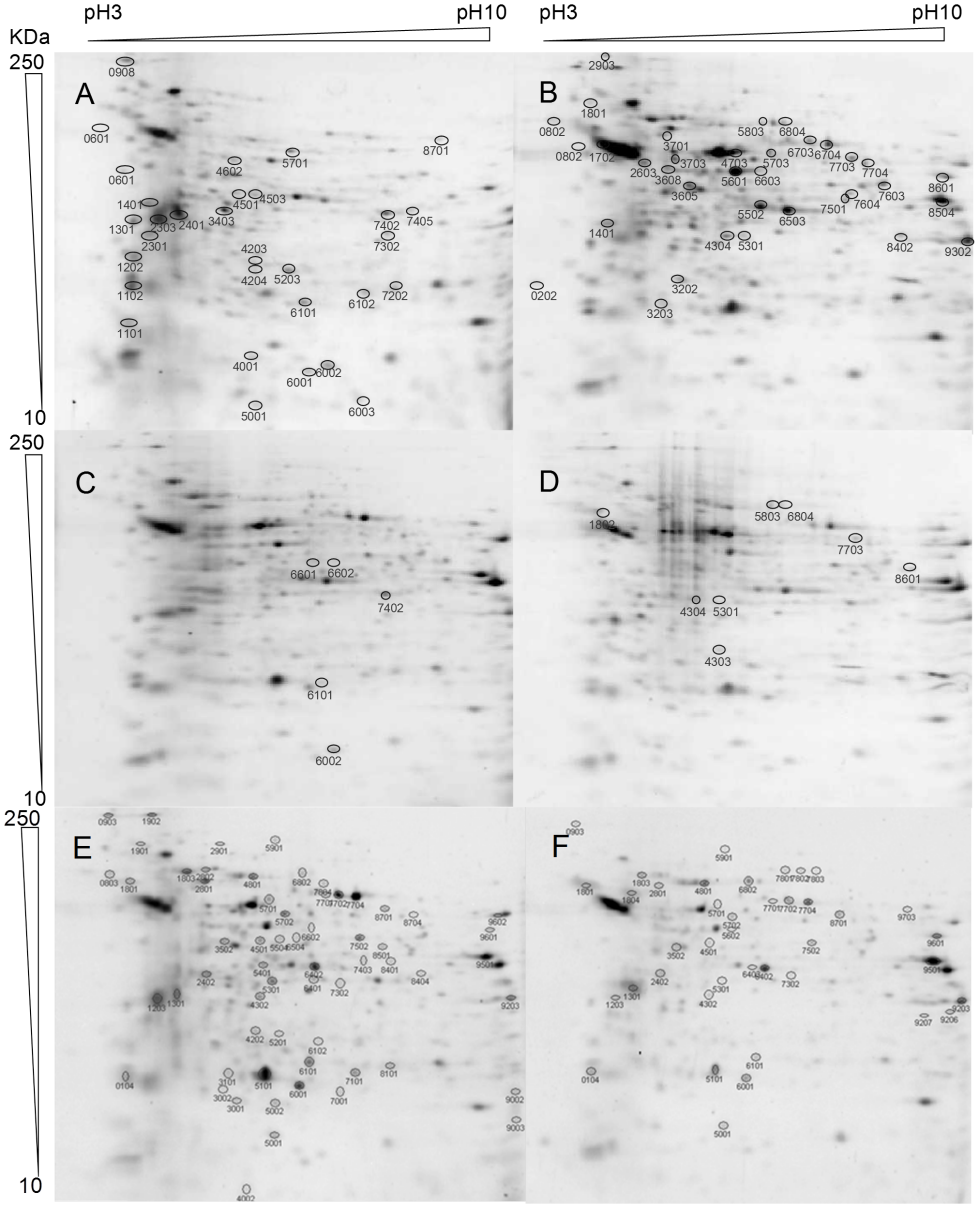
2DE of total protein extracts throughout the growth curve and of the PNA^+^ and PNA^-^ subpopulations of *C. fasciculata* choanomastigotes. 2DE of 50 µg of total protein extracts of *C. fasciculata* choanomastigotes at (A) early logarithmic, (B) mid logarithmic, (C) late logarithmic and (D) stationary phase. (E) PNA^-^ and (F) PNA^+^ subpopulations. One out of three replicates is shown for each phase/subpopulation. IEF was performed in a non-linear 3–10 pH interval. Complete spot names include Cf (A–D) or Cf_p_ (E and F) preceding the spot numbers (see Tables 1–3).

Choanomastigote cultures reached the stationary phase within 3 days (Fig. 1B), twice as fast as *Leishmania* spp. promastigotes. Similar growth kinetics of *C. fasciculata* choanomastigote clones has been reported [20]. Total protein of 5×10^8^ choanomastigotes was extracted at early logarithmic (day 1), mid-logarithmic (day 2), late logarithmic/early stationary (day 3) and stationary phase (day 4). In addition, protein extracts were successfully obtained from the PNA^+^ and PNA^-^ subpopulations within the cultures in stationary phase. Protein concentrations were comprised between 4 and 9 µg/µl and this was confirmed by PAGE-SDS as described [14]. After 2DE separations, normalization with the *Total Quantity in Valid Spots* algorithm and manual check of all the spots, 136 changes in abundance of proteins were detected throughout the four time points of the growth curve. Some proteins showed significant differences in abundance in more than one time point of the choanomastigote growth curve. The cut-off values were: ratio to day 1, R≥1.7 or≤0.6 within the significance level inferred with Student's t test (p<0.05). Of these, 63 spots that correspond to 83 differences in abundance (Fig. 2A–D, Table 1) were excised from the 2DE gels as they were suitable to be identified by MALDI-TOF/TOF. Therefore, 10 proteins are differentially expressed at two of the time points compared. We also analyzed 43 spots containing constantly expressed proteins (Table 2), as well as 67 spots differentially expressed between the PNA^-^ and PNA^+^ subpopulations within the stationary phase culture (Fig. 2E and F, Table 3). All proteins could be identified when MASCOT searches were performed against the reference genome sequence of *C. fasciculata* (Tables 1–3) when there was sufficient amount for identification, whereas a total of 20 constantly expressed proteins (Table S2 in S1 File), 41 differentially expressed proteins in the growth curve (Table S1 in S1 File) and 30 proteins with different abundance between PNA^+^ and PNA^-^ choanomastigotes (Table S3 in S1 File) could be identified against the *NCBInr* database, which is 53.4% of the proteins analyzed by MALDI-TOF/TOF mass spectometry. Most of the identifications (73.9%) were consistent between the two databases and most of those successfully performed with the *NCBInr* database (63.9%) had the highest MASCOT scores for orthologue proteins of the genus *Leishmania*, whereas only 9.3% of them matched with a *Trypanosoma* spp. orthologue (Tables S1–S3 in S1 File). This is additional evidence for the hypothesis supporting very close evolutionary relationship between *Leishmania* spp. and *Crithidia* spp [6], [7], [8], [9]. Also, only 9.3% matched with *Crithidia* spp., as very few genes had been identified in this organism prior to the release of the reference genome sequence (Tables S1–S3 in S1 File). Sometimes, different spots represent the same type of protein. This may be due to the presence of different isoforms, post-translational modifications or protein aggregation at the conditions assayed. In the next sections, we refer to these possibilities using the term variant of a given protein.

**Table 1:** Differentially regulated proteins throughout the growth curve of *C. fasciculata* choanomastigotes.

Spot	Protein	TriTryp Id.	MW (KDa)	*MASCOT* score (p<0.05)	Ratio (p<0.05)		
					d2:d1	d3:d1	d4:d1
Cf0202	Thiol-dependent reductase 1, putative	CfaC1_35_0470	26.5	187	_	0.01	_
Cf0601	Mitotubule-associated protein Gb4, putative	CfaC1_33_0870	45.0	48*	0.58	_	_
Cf0908	Mitotubule-associated protein, Gb4, putative	CfaC1_33_0870	170.0	455	0.50	_	_
Cf1101	60S ribosomal protein L21, putative	CfaC1_17_0510	17.9	30*	0.01	_	_
Cf1102	Eukaryotic initiaton factor 5a, putative	CfaC1_28_1000	22.2	44*	0.58	_	_
Cf1103	Eukaryotic initiation factor 5a, putative	CfaC1_28_1000	23.6	271	0.48	_	_
Cf1202	Hypothetical protein, conserved	CfaC1_11_0220	26.8	116	0.44	_	_
Cf1301	Translation elongation factor 1β, putative	CfaC1_30_1700	32.8	126	0.58	_	_
Cf1801	Peroxisomal targeting signal-1 receptor	CfaC1_31_1260	92.6	75	_	0.32	_
Cf1802	Unnamed protein product	Cfa_19_1120	70.4	144	_	_	1.72
Cf2303	Hypothetical protein	CfaC1_16_2100	33.6	90	0.24	_	_
Cf2401	Unspecified product	CfaC1_AODS01004023_0010	35.0	89	0.21	_	_
Cf2603	Thiol-dependent reductase 1, putative	CfaC1_35_0470	53.9	187	3.18	_	_
Cf2903	Hypothetical protein, conserved	CfaC1_22_0410	227.4	33*	89.60	0.18	_
Cf3202	Phosphomannomutase, putative	CfaC1_30_2340	27.2	380	1.78	_	_
Cf3203	Tryparedoxin peroxidase (*C. fasciculata*)	CfaC1_10_1430 (gi3851500)	24.1	138	_	3.28	_
Cf3401	Transaldolase B, putative	CfaC1_17_0910	36.1	299	0.46	_	_
Cf3403	CACK protein (*C. fasciculata*), activated protein kinase C receptor (LACK), guanine nucleotide-binding protein β subunit-like	CfaC1_26_3810 (gi3132790)	36.5	210	0.58	_	_
Cf3605	Hypothetical protein	CfaC1_07_1350	46.9	137	2.34	_	_
Cf3608	Unspecified protein product (enolase orthologue)/Oxidoreductase-like protein	CfaC1_AODS01003826_0010/CfaC1_22_0890	51.1	173	1.73	_	_
Cf3701	Chaperonin hsp60, mitochondrial precursor	CfaC1_30_2420	67.5	377	_	0.01	_
Cf3703	Vacuolar ATP synthase subunit B	CfaC1_26_3200	55.8	426	8.41	_	_
Cf4001	Unspecified protein	CfaC1_AODS01003826_0010	15.9	173	0.58	_	_
Cf4204	SNARE protein, putative	CfaC1_19_0830	26.0	34*	0.03	_	_
Cf4303	Putative GTP-binding protein	CfaC1_28_1830	29.1	164	_	_	1.76
Cf4304	Aldose 1-epimerase-like protein, putative	CfaC1_33_4840	34.5	330	1.84	_	1.93
Cf4501	Unspecified product (enolase orthologue)	CfaC1_AODS01003826_0010	41.0	194	0.50	_	_
Cf4503	Unspecified product (Enolase ortologue)	CfaC1_AODS01003826_0010	41.0	357	0.56	_	_
Cf4602	Glutamate dehydrogenase, putative	CfaC1_26_3700	50.2	319	2.12	_	_
Cf4703	Pyruvate/indol pyruvate carboxylase, putative	CfaC1_33_5440	59.6	333	458.61	_	_
Cf5101	Tryparedoxin peroxidase	CfaC1_10_1430 (gi3851500)	12.9	186	0.02	_	_
Cf5203	Hypothetical protein	CfaC1_19_1320	26.5	154	0.58	_	_
Cf5301	Unspecified product (Glycosomal malate dehydrogenase orthologue)	CfaC1_AODS01001854_0010	34.2	106	2.15	_	2.85
Cf5502	Alcohol dehydrogenase	CfaC1_06_0480	40.87	642	_	0.58	_
Cf5601	Unspecified product (enolase orthologue)	CfaC1_AODS01003826_0010	51.2	728	1.84	_	_
Cf5701	Hypothetical protein, conserved	CfaC1_30_4240	55.9	279	0.58	_	_
Cf5703	Pyruvate kinase	CfaC1_24_1930	59.3	77	3.94	_	_
Cf5803	Transketolase	CfaC1_30_4190	75.7	193	28.65	35.21	39.96
Cf6001	ADF/Cofilin, putative	CfaC1_14_1580	15.0	235	0.57	_	_
Cf6002	Small myristoylated protein-1, putative	CfaC1_19_1600	15.4	233	0.50	1.70	_
Cf6003	Elongation factor 2, putative	CfaC1_30_0260	12.9	234	0.01	_	_
Cf6101	Fe-superoxide dismutase	CfaC1_32_2350	21.8	321	0.58	1.72	_
Cf6102	NADP-dependent alcohol dehydrogenase, putative	CfaC1_22_0610	23.4	95	0.37	_	_
Cf6503	NADP-dependent alcohol dehydrogenase	CfaC1_22_0610	40.4	267	1.93	_	_
Cf6602	Mitotubule-associated protein Gb4, putative	CfaC1_33_0870	45.6	134	_	11.1	_
Cf6603	Unspecified product (enolase orthologue)	CfaC1_AODS01003826_0010	51.2	230	80.53	_	_
Cf6703	Catalase	CfaC1_30_0050	64.6	125	3.44	_	_
Cf6704	Catalase	CfaC1_30_0050	63.3	189	3.26	_	_
Cf6804	Transketolase	CfaC1_30_4190	75.6	125	57.40	_	72.40
Cf7202	NADP-dependent alcohol dehydrogenase, putative	CfaC1_22_0610	24.2	125	0.51	_	_
Cf7302	Fructose-1,6-bisphosphate aldolase, putative	CfaC1_30_1480	32.9	418	0.23	_	_
Cf7402	Fructose-1,6-bisphosphate aldolase, putative	CfaC1_30_1480	36.8	407	0.35	3.02	_
Cf7405	Fructose-1,6-bisphosphate aldolase, putative	CfaC1_30_1480	37.6	278	0.29	_	_
Cf7501	Phosphoribosylpyrophosphate synthetase, putative, phosphoribosyl transferase, putative	CfaC1_04_0370	42.7	60	2.6	_	_
Cf7603	3-ketoacyl-CoA thiolase, putative	CfaC1_22_1180	47.4	607	1.74	_	_
Cf7604	Pyruvate dehydrogenase component E1 a-subunit, putative	CfaC1_21_1950	44.4	143	57.93	_	_
Cf7703	Succinyl-CoA:3-ketoacid-coenzyme A transferase, mitochondrial precursor, putative	CfaC1_04_0870	56.7	189	2.22	_	2.14
Cf7704	Dihydrolipoamide dehydrogenase, putative	CfaC1_32_4840	54.8	507	2.72	_	_
Cf8402	Malate dehydrogenase	CfaC1_33_1570	34.9	663	2.38	_	_
Cf8504	Fructose-1,6-bisphosphate aldolase, putative	CfaC1_30_1480	42.8	594	2.93	_	_
Cf8601	Hexokinase	CfaC1_31_0400	49.3	683	4.62	_	4.83
Cf8701	Hypothetical protein	CfaC1_32_1140	62.5	349	0.50	_	_
Cf9302	Unspecified product (glycosomal malate dehydrogenase orthologue)	CfaC1_AODS01001854_0010	33.7	379	1.99	_	_

Estimated MW, pI, MASCOT scores (*non-significant) and ratios to day 1 are provided. Only spots with statistically significant ratios (p<0.05) over 1.7 or under 0.6 were picked and analyzed and are shown in this table. As a consequence, hyphens in the columns containing ratios do not necessarily indicate lack of differential abundance, because there are also cases of lack of statistical significance of ratios indicating over- or under-expression. Identifications were performed against the *C. fasciculata* genome sequence released in the TriTryp database.

**Table 2:** Constantly expressed proteins throughout the growth curve of *C. fasciculata* choanomastigotes.

Spot	Protein	TriTrypDB Id.	MW (KDa)	pI	MASCOT score (p<0.05)
Cf1001	ATP-dependent RNA-helicase	CfaC1_32_0820	17.91	4.5	28*
Cf1602	META domain-containing protein	CfaC1_15_1260	52.82	4.6	613
Cf1904	Hypothetical protein	CfaC1_24_0880	105.02	4.4	569
Cf2201	Hypothetical protein, conserved	CfaC1_33_5560	27.96	5.2	38*
Cf2702	Hypothetical protein	CfaC1_16_2100	60.31	4.7	50*
Cf2703	ATG8/AUT7/APG8/PAZ2	CfaC1_19_0910	57.83	5.0	28*
Cf2804	Unnamed protein product	CfaC1_24_2570	91.86	5.0	122
Cf2805	Hypothetical protein	CfaC1_26_3570	76.77	5.3	150*
Cf2901	Unspecified protein	CfaC1_KB217687_0080	171.73	4.7	32*
Cf4201	Proteasome activator protein pa26, putative	CfaC1_24_2680	25.11	5.7	337
Cf4202	Short chain dehydrogenase, putative	CfaC1_31_1020	27.95	5.7	132
Cf4301	Biotin/lipoate protein ligase-like protein	CfaC1_34_1660	28.88	5.7	109
Cf4302	Prostaglandin f2α synthase/D-arabinose dehydrogenase, putative	CfaC1_34_3900	33.04	5.7	114
Cf4401	Thymine 7-hydroxylase, putative	CfaC1_14_1860/70	36.76	5.8	60
Cf6202	Iron superoxide dismutase, putative	CfaC1_25_0490	25.01	6.1	55
Cf6401	Coproporphyrinogen III oxidase	CfaC1_24_0220	35.33	6.1	687
Cf6402	Methylthioadenosine phosphorylase, putative	CfaC1_05_0930	37.29	6.1	101
Cf6501	NADP-dependent alcohol dehydrogenase	CfaC1_22_0610	40.91	6.0	181
Cf6502	Branched-chain amino acid aminotransferase, putative	CfaC1_27_2440	42.86	6.1	184
Cf6506	Arginase, putative	CfaC1_31_1380	39.73	6.3	123
Cf6507	Chaperone protein DNAJ, putati ve/Unspecified product	CfaC1_32_4400/CfaC1_AODS01001585_0010	42.47	6.3	40/40*
Cf6708	Aldehyde dehydrogenase, mitocondrial precursor, putative	CfaC1_28_1490	55.55	6.1	256
Cf6901	Aconitase	CfaC1_21_0760	104.44	6.4	454
Cf7002	Nucleoside diphosphate kinase	CfaC1_32_3660	16.09	6.7	295
Cf7201	Ribulose phosphate 3-epimerase, putative	CfaC1_31_3770	27.35	6.4	85
Cf7204	ATP synthase, putative	CfaC1_35_2640	27.71	7.5	176
Cf7301	RNA-binding protein	CfaC1_31_2330	37.67	6.5	433
Cf7601	Aldose 1-epimerase-like protein, putative	CfaC1_24_2950	46.66	6.4	148
Cf7602	Phosphoglycerate kinase B, cytosolic	CfaC1_23_0180	46.54	6.5	621
Cf7705	Glycosomal phosphoenolpyruvate carboxykinase, putative	CfaC1_27_2690	62.20	6.7	191
Cf8102	Cyclophilin A, putative	CfaC1_28_1250	19.39	8.6	356
Cf8104	Cyclophilin 4, putative	CfaC1_04_0140	22.57	9.1	252
Cf8202	Triose phosphate isomerase	CfaC1_27_0920	24.89	7.5	295
Cf8203	Triose phosphate isomerase (*L. braziliensis*)	CfaC1_27_0920	25.02	8.3	126
Cf8204	RNA-binding protein, putative, UPB2	CfaC1_28_0710	24.03	9.1	103
Cf8301	Hypothetical protein	CfaC1_18_0790	31.53	8.7	125
Cf8302	Succinyl-CoA synthetase α-subunit, putative	CfaC1_02_0750	33.63	8.7	124
Cf8501	Aldose 1-epimerase-like protein, putative	CfaC1_24_2950	41.01	8.0	509
Cf8502	Hypothetical protein, conserved/Hypothetical protein, conserved	CfaC1_35_4400/CfaC1_33_5170	39.25	8.3	36/36*
Cf8702	Poly(A)-binding protein 2	CfaC1_31_0480	68.60	9.0	352
Cf9301	Hypothetical protein, conserved (gMDH)	CfaC1_13_1170	31.77	9.2	272
Cf9401	Elongation factor 1α, putative	CfaC1_15_0180	36.35	9.3	149
Cf9601	Elongation factor 1α, partial	CfaC1_15_0180	53.84	9.2	165

Estimated MW, pI and MASCOT scores (*non-significant) are provided. Identifications were performed against the *C. fasciculata* genome sequence released in the TriTryp database.

**Table 3:** Differential abundance of proteins between the PNA^+^ and PNA^-^ subpopulations of *C. fasciculata* choanomastigotes in stationary phase of axenic culture.

Spot	Protein	TriTryp DB Id.	MW (KDa)	pI	*MASCOT* score (p <0.05)	Ratio (PNA^+^):(PNA^-^) (p <0.05)
Cf_p_0104	Eukaryotic initiation factor 5a	CfaC1_28_1000	20.52	3.5	70	0.44
Cf_p_0803	Mitotubule-associated protein Gb4, putative	CfaC1_33_0870	73.19	3.4	388	0.02
Cf_p_0903	Mitotubule-associated protein Gb4, putative	CfaC1_33_0870	130.8	3.3	161	0.29
Cf_p_1203	Hypothetical protein	CfaC1_16_2100	35.48	3.8	102	0.17
Cf_p_1803	Hypothetical protein	CfaC1_26_3570	86.91	4.7	125	0.41
Cf_p_1901	Hypothetical protein, conserved	CfaC1_24_0880	142.13	4.3	354	0.14
Cf_p_2402	Hypothetical protein/Unspecified product	CfaC1_27_1220/CfaC1_AODS01003272_0020	34.22	5.2	24/23	0.57
Cf_p_2801	2,3-bisphosphoglycerate-independent phosphoglycerate mutase, putative	CfaC1_35_3710	76.42	5.0	483	0.40
Cf_p_2802	Carboxylase, putative	CfaC1_12_0120	88.35	5.1	175	0.15
Cf_p_2901	Oligopeptidase B, putative	CfaC1_12_1200	92.75	5.4	59	0.03
Cf_p_3001	NADP-dependent alcohol dehydrogenase, putative	CfaC1_22_0610	18.46	5.5	40*	0.01
Cf_p_3002	Unspecified product	CfaC1_19_1390	19.12	5.4	22*	0.07
Cf_p_3101	Tryparedoxin peroxidase (*C. fasciculata*)	CfaC1_10_1430 (gi385150)	22.36	5.4	169	0.01
Cf_p_4002	Nucleoside diphosphate kinase b	CfaC1_32_3660	10.52	5.5	38*	0.03
Cf_p_4202	GTP-binding protein	CfaC1_28_2830	23.10	5.6	133	0.03
Cf_p_4302	Aldose 1-epimerase-like protein, putative	CfaC1_33_4840	28.68	5.6	82	0.52
Cf_p_4501	Unspecified product/Unspecified product	CfaC1_AODS01001347_0030/CfaC1_AODS01003093_0020	42.76	5.6	60/60	0.48
Cf_p_5001	Endoribonuclease L-PSP (pb5)	CfaC1_22_0370	14.23	5.9	82	0.56
Cf_p_5002	60S ribosomal protein L37a, putative	CfaC1_16_2060/CfaC1_30_2280	18.15	5.7	25/25*	0.02
Cf_p_5101	Tryparedoxin peroxidase (*C. fasciculata*)	CfaC1_10_1430 (gi385150)	20.46	5.7	174	0.46
Cf_p_5201	Insufficient amount for identification	-	27.17	5.8	-	0.09
Cf_p_5301	Unnamed protein product (enolase orthologue)	CfaC1_AODS01003826_0010	41.26	5.7	442	0.33
Cf_p_5401	Nucleoside phosphorylase-like protein, putative	CfaC1_18_1820	36.22	5.6	57	0.03
Cf_p_5504	Methionine aminopeptidase 2, putative	CfaC1_16_0810	43.54	5.8	29*	0.04
Cf_p_5701	Hypothetical protein	CfaC1_05_0410	60.28	5.7	36*	0.31
Cf_p_5702	Hypothetical protein, conserved	CfaC1_30_4240	47.91	5.9	170	0.57
Cf_p_5901	Elongation factor 2, putative	CfaC1_30_0260	171.5	5.8	59	0.54
Cf_p_6001	Fe-superoxide dismutase	CfaC1_32_2350	19.25	6.0	356	0.59
Cf_p_6101	Iron superoxide dismutase	CfaC1_25_0490	23.09	6.0	96	0.56
Cf_p_6102	Insufficient amount for identification	-	25.23	6.0	-	0.06
Cf_p_6401	Coproporphyrinogen III oxidase	CfaC1_24_0220	38.52	6.1	533	0.28
Cf_p_6504	Hypothetical protein	CfaC1_19_0530	44.34	5.9	25*	0.05
Cf_p_6602	Insufficient amount for identification	-	46.81	6.0	-	0.07
Cf_p_7001	Hypothetical protein, conserved/Hypothetical protein, conserved	CfaC1_35_5730/40	19.87	6.2	36/35	0.03
Cf_p_7101	NADP-dependent alcohol dehydrogenase, putative	CfaC1_22_0610	23.37	6.3	25*	0.01
Cf_p_7701	Catalase, putative	CfaC1_30_0050	59.30	6.1	83	0.03
Cf_p_7403	Hypothetical protein	CfaC1_30_0060	36.21	6.3	130	0.03
Cf_p_7502	Aldose 1-epimerase-like protein	CfaC1_24_2950	53.96	6.3	169	0.64
Cf_p_7702	Catalase	CfaC1_30_0050	87.42	6.2	159	0.53
Cf_p_7704	Catalase	CfaC1_30_0050	87.11	6.3	215	0.36
Cf_p_7804	Fumarate hydratase, putative	CfaC1_25_2280	98.56	6.1	95	0.06
Cf_p_8101	Hypothetical protein, conserved	CfaC1_28_2780	24.22	6.5	21*	0.08
Cf_p_8401	Hypothetical protein	CfaC1_26_0530	39.90	6.5	24*	0.04
Cf_p_8404	Insufficient amount for identification	-	36.55	7.0	-	0.05
Cf_p_8501	Insufficient amount for identification	-	41.22	6.3	-	0.04
Cf_p_8704	Insufficient amount for identification	-	52.18	6.4	-	0.09
Cf_p_9002	Insufficient amount for identification	-	20.12	9.5	-	0.01
Cf_p_9003	Insufficient amount for identification	-	16.98	9.5	-	0.02
Cf_p_9602	Elongation factor 1α, putative	CfaC1_15_0180	71.43	9.3	447	0.01
Cf_p_1301	Sphingosine kinase A, B, putative	CfaC1_08_0670	36.09	4.6	32*	3.31
Cf_p_1801	Dipeptidylcarboxypeptidase, putative	CfaC1_27_1750	70.35	3.5	38*	12.43
Cf_p_1804	Hypothetical protein	CfaC1_13_1510	71.01	4.9	38*	503.6
Cf_p_3502	TATE DNA transposon	CfaC1_19_2160	49.69	5.5	22*	1.96
Cf_p_4801	Hypothetical protein	CfaC1_50_0070	78.23	5.7	38*	3.31
Cf_p_5602	Insufficient amount for identification	-	56.13	6.9	-	40.85
Cf_p_6402	NADP-dependent alcohol dehydrogenase, putative	CfaC1_22_0610	44.01	6.5	177	1.77
Cf_p_6403	NADP-dependent alcohol dehydrogenase, putative	CfaC1_22_0610	44.01	6.5	39*	98.62
Cf_p_6802	Transketolase, putative	CfaC1_30_4190	81.44	6.4	109	2.69
Cf_p_7302	Fructose-1,6-bishosphate aldolase, putative	CfaC1_30_1480	39.05	6.2	270	1.92
Cf_p_7801	Hypothetical protein, conserved	CfaC1_18_1860	90.58	6.6	52*	59.04
Cf_p_8701	Succinyl-CoA: 3-ketoacid-CoA transferase, mitochondrial precursor, putative	CfaC1_04_0870	75.28	6.5	129	2.01
Cf_p_9203	Unspecified product (glycosomal malate dehydrogenase orthologue)	CfaC1_AODS01001854_0010	35.74	9.6	188	3.16
Cf_p_9501	Fructose-1,6-bisphosphate aldolase, putative	CfaC1_30_1480	48.93	9.2	526	1.89
Cf_p_9206	Insufficient amount for identification	-	31.88	9.4	-	50.78
Cf_p_9207	Insufficient amount for identification	-	31.29	8.5	-	49.33
Cf_p_9601	Hexokinase	CfaC1_31_0400	61.24	9.2	611	7.26
Cf_p_9703	Insufficient amount for identification	-	64.73	8.3	-	0.68

Estimated molecular weights, pI, MASCOT scores (*non-significant) and PNA^+^/PNA^-^ ratios. Only spots with statistically significant ratios (p<0.05) over 1.7 or under 0.6 were picked and analyzed and are shown in this table. Identifications were performed against the *C. fasciculata* genome sequence released in the TriTryp database.

### Peanut lectin agglutination capability of choanomastigotes

It is known that certain species of *Leishmania* agglutinate with PNA and a non-agglutinating subpopulation can be isolated [10], [11]. This is the first time that the agglutination properties of *Crithidia* spp. choanomastigotes with PNA have been assayed and the outcome has been a noticeable agglutination capability and the isolation of a non-agglutinating population in stationary phase. These findings support a modification of the *Crithidia* spp. lipoarabinogalactans (LAG) at the end of the growth curve analogous to that taking place in the lipophosphoglycan (LPG) of *Leishmania* spp., even when the structure of the LAG [21], the surface molecules that presumably agglutinate with the lectin in this genus, is quite different to the leishmanial LPG. The biological roles of the respective agglutinating surface molecules involved are probably different in these organisms given the differences in their life cycles. *L. major* and *L. infantum* promastigotes are able to agglutinate with PNA and the non-agglutinating subpopulations are more infective and lead to higher infection rates than the agglutinating ones, yielding more infected phagocytes and amastigotes per infected cell [10], [11]. Promastigotes attach to the gut epithelium by the LPG to maintain infection during bloodmeal excretion and only with differentiation signals as starvation, a developmental process ultimately leading to metacyclic promastigotes is triggered (reviewed in [22]). The differentiation process is widely recognized to be mimicked in axenic culture, where starvation also takes place when promastigotes reach the stationary phase.

The change in LAG composition of *C. fasciculata* is likely due to developmental processes according to the findings described herein (see below) but does not provide evidence to be associated to any process related with infectivity. In fact, the next step in the life cycle of this parasite is differentiation to the extracellular amastigote stage that attaches to the gut epithelium of the insect host.

### Changes in abundance of proteins involved in glucid catabolism and the pentose phosphate pathway

**Figure 3:**
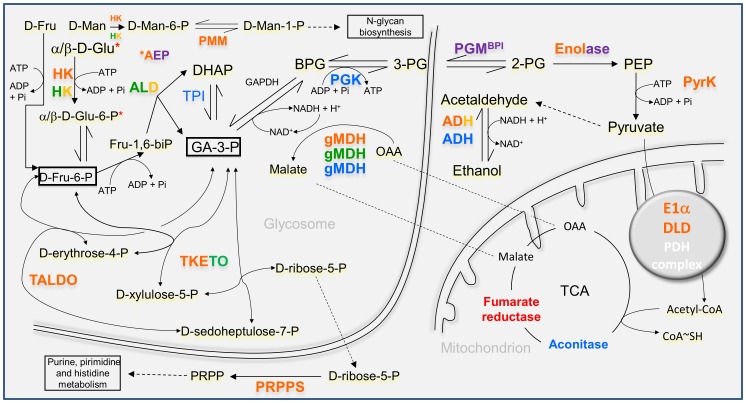
Differentially expressed proteins related with carbohydrate metabolic processes in *C. fasciculata* choanomastigotes. Functional connection of differentially expressed enzymes involved in glucid metabolic processes. Legend: proteins/protein variants in blue are constitutively expressed throughout the growth curve; proteins/protein variants in orange are up-regulated at day 1 or 2 (logarithmic phase); proteins/variants in green are up-regulated at day 3 or 4 (late logarithmic/stationary phase); proteins in red are up-regulated in PNA^+^ choanomastigotes; proteins in purple are up-regulated in PNA^-^ choanomastigotes.

According to proteome profiling, glycolysis is more active in early and mid logarithmic phase (Fig. 3, Table 1), when several protein variants alternate. In fact, two aldolase (ALD) and two enolase variants are up-regulated at day 1 but at the second day, their expression levels decay and are replaced by distinct ones, respectively one ALD and two enolases. Additionally, an hexokinase (HK) variant, the pyruvate kinase (PyrK), a putative and a glycosomal malate dehydrogenase (MDH) and the components of the pyruvate dehydrogenase complex (PDH) dihydrolipoamide dehydrogenase (DLD) and E1α are more abundant at day 2 (mid logarithmic phase), which suggests higher activity of hexose catabolic processes and malate shuttles, provided that most of the glucolytic reactions take place in the glycosome of these organisms (reviewed in [23]). In fact, other monosaccharides may be increasingly utilized by choanomastigotes in mid logarithmic phase as additional carbon and energy sources and/or to provide precursors for the biosynthesis of glycans. This is suggested on the basis of the wide substrate specificity of the HK and the up-regulation of the phosphomannomutase (PMM) and the aldose 1-epimerase (AEP). The PMM is involved in the biosynthesis of N-glycans providing manose-1-phosphate, as the reaction is reversible. The AEP is also up-regulated at the stationary phase, especially in PNA^-^ choanomastigotes (Tables 1 and 3). As highlighted in Fig. 3, the up-regulation of these ALD variants may yield high levels of glyceraldehyde-3-phosphate not only for the subsequent glucolytic reactions but also for the pentose-phosphate shunt, provided the up-regulation of the transaldolase B (TALDO) at early logarithmic phase (day 1) and the transketolase (TKETO) at mid logarithmic and stationary phase. These proteins are related functionally with the phosphoribosyl pyrophosphate synthase (PRPPS), which is more abundant at day 2. These findings are indicative of maximum activity of the glucolytic pathway at mid logarithmic phase providing energy and essential precursors of certain amino acids, ribonucleotides and derived coenzymes. By contrast, the highest expression levels of genes involved in glucolysis are found in *L. infantum* promastigotes in stationary phase [24] and differential regulation of genes involved in the pentose phosphate shunt has not been detected up to date in these pathogenic trypanosomatids.

The up-regulation of two alcohol dehydrogenases (ADH) at early logarithmic phase is also related with sugar catabolism, as a relative inefficiency of the respiratory process and the finding of subproducts as ethanol and lactate were described in trypanosomatids (reviewed in [25]). Alternation in up-regulation between ADH variants has been detected between PNA^+^ and PNA^-^ choanomastigotes (Table 3), and there are also constitutive and differentially regulated variants of ADH and MDH.

In certain cases, only proteins that catalyze irreversible reactions and/or control the kinetics of the metabolic process are increasingly abundant, whereas those catalyzing reversible and non-limiting steps are constitutively expressed. This is the case of the expression profile of glycolytic enzymes in *C. fasciculata* choanomastigotes as the triose phosphate isomerase (TPI) and the phosphoglycerate kinase (PGK) (Fig. 3, Table 2) are constitutively expressed unlike others mentioned above. Conversely, an *L. infantum* orthologue of the PGK reaches its highest abundance in stationary phase promastigotes at the transcript level [24]. The aconitase is up-regulated in the promastigote stage with respect to amastigotes in *L. infantum* [26] and the opposite profile was observed in *L. donovani* [27], but the *C. fasciculata* orthologue is constitutively expressed in the choanomastigote stage.

Several enzymes related with sugar metabolism are also differentially regulated between PNA^+^ and PNA^-^ choanomastigotes within the stationary phase. The PGM^BPI^ and the enolase are over-expressed, suggesting that only the second part of glycolysis is more active in PNA^-^ choanomastigotes in a bisphosphoglycerate-independent manner. As a difference with mammalian organisms, the trypanosomatid genomes encode the PGM^BPI^, which suggests that this protein is a good drug target [28]. Interestingly, this enzyme is differentially regulated between different life cycle stages in *L. infantum* [24] and also in *C. fasciculata* according to the analysis described herein. The expression patterns of the HK, ALD and TKETO are the opposite to those of the PGM^BPI^ and the enolase. Consequently, the expression profile of the glycolytic and the pentose phosphate pathway are the same in the PNA^+^ subpopulation and the whole population in stationary phase (Table 3). The finding is coherent as this is the major subpopulation at that growth phase.

The heme biosynthetic enzyme coproporphyrinogen (III) oxidase (C(III)O) is up-regulated in PNA^-^ *C. fasciculata* choanomastigotes (Table 3) whereas it is constitutively expressed throughout the growth curve (Table 2). Differential expression was not observed during axenic growth of *L. infantum* promastigotes either and it was revealed that C(III)O is up-regulated in intracellular amastigotes [24] and axenic amastigotes obtained by temperature and pH shift [29]. The heme group is necessary for a variety of cellular functions in *Leishmania* spp., including not only the electron transport chain, but also the catalase, which is also over-expressed in the PNA^-^ subpopulation (see below).

### Changes in abundance of proteins involved in lipid metabolism

Fatty acid biosynthesis, ketone body degradation, β-oxidation of fatty acids and/or branched chain amino acid degradation is probably more activated at day 2 because a putative 3-ketoacid-CoA thiolase (KAT) is up-regulated. This may be linked with the up-regulation of the succinyl-CoA:3-ketoacid-CoA transferase (SCAT), which suggests the utilization of ketone bodies in *C. fasciculata* mid logarithmic phase choanomastigotes, which is of unknown meaning in trypanosomatids. The existence of ketone bodies in trypanosomatids was tested by ^1^H NMR in *L. donovani* axenic amastigotes [30]. These molecules are probably mere intermediate metabolites in these organisms. Given that the SCAT catalyzes the split of acetoacetate into two acetyl-CoA molecules, a probable explanation for its up-regulation may be that it is involved in the last step of Leu degradation, as well as KAT up-regulation suggests a role in Ile catabolism and the DHL in degradation of all branched chain amino acids. Conversely, thiolases reach their highest expression levels in stationary phase promastigotes in *L. infantum* [24]. The SCAT is down-regulated in the PNA^-^ choanomastigote subpopulation, which indicates a decrease throughout choanomastigote development taken together with the results obtained for the growth curve.

### Changes in abundance of proteins involved in gene expression regulation and signal transduction

A decreased translational elongation rate is expected throughout the growth curve of choanomastigotes, as the abundance of the eukaryotic translation initiation factor 5a (eIF5a), the translation elongation factor 1β (EF1β) and the elongation factor 2 (EF2) decreases. This is likely due to the higher metabolic activity and faster growth in logarithmic phase choanomastigotes. However, other proteins involved in gene expression regulation that are differentially abundant in *L. infantum* promastigotes [24], [31] are constitutively expressed conversely in *C. fasciculata* choanomastigotes: the EF1α, a poly(A) binding protein (PABP2), two RNA-binding proteins (RNA bp) and an ATP-dependent RNA helicase (Table 2). These proteins may be involved in developmental processes rather than in growth in *Leishmania* spp. promastigotes. A similar hypothesis can be posed to explain the up-regulation of the eIF5a, the EF1α, the EF2 and the endoribonuclease L-PSP in the PNA^-^ choanomastigotes, as this minor subpopulation was isolated from culture in stationary phase, where growth conditions would not explain an increase on the translation rate. The transcript levels of PABP are also higher in *L. infantum* PNA^-^ promastigotes, but those of the EF1α were significantly lower, thus being the expression profile the opposite [11]. In fact, three variants of the EF1α protein are more abundant in stationary phase promastigotes, where the PNA^+^ promastigote subpopulation is by far the more represented [14]. Future in depth analysis of these differences between both parasites may aid to explain why their developmental processes are different.

As for protein folding, only a variant of the hsp60 chaperonin is more abundant in early logarithmic phase choanomastigotes, whereas other variant of this protein, a DnaJ domain-containing protein, two cyclophilins and the adenosine kinase domain-containing nucleoside diphosphate kinase b (Ndkb) are constitutively expressed throughout the growth curve of axenic choanomastigotes. These findings also contrast with results found in *L. infantum* promastigotes [11], [14], [24], [31]. To put an example, a cyclophilin is up-regulated in stationary phase promastigotes in *L. infantum*.

**Figure 4:**
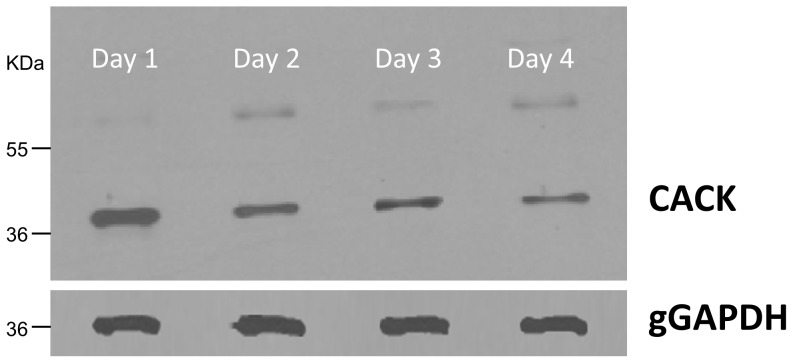
CACK is up-regulated in logarithmic phase choanomastigotes. Detection and differential expression analysis of CACK in 20 µg total protein extracts by Western blot with 1∶500 polyclonal antibody against the LACK analogue throughout the choanomastigote growth curve. The ∼60 KDa band presumably contains dimeric CACK aggregates (González-Aseguinolaza et al., 1999). gGAPDH is the protein of reference (dilution 1∶10,000 of the monoclonal antibody).

Regarding intracellular signalling, the *C. fasciculata* analogue (CACK) (gi3132790; CfaC1_26_3810) of the receptor of the activated protein kinase C (RACK) is up-regulated in early logarithmic phase choanomastigotes (day 1) (Fig. 2, Table 1). This expression profile has been confirmed by Western blot (Fig. 4, Figures S1 and S2 in S2 File), which has revealed the progressive descent of CACK abundance throughout the choanomastigote growth curve. The leishmanial orthologue LACK, an antigenic protein that partially protects against canine leishmaniasis [32], [33], [34], is located in the particulate fraction of the cytoplasm near the plasma membrane. LACK is up-regulated in *L. infantum* amastigotes but constantly expressed in promastigotes [18], [24]. Consequently, the CACK/LACK expression patterns in the motile stages of *C. fasciculata* and *L. infantum* are different, what suggests different roles in proliferation and differentiation in their respective life cycles. PKCs are translocated by their receptor (RACK) to different intracellular sites [35] and activated via phospholipase C or Ca^2+^. PKCs and RACKs are involved in a variety of characterized signal transduction cascades in mammals [36]. However, their role in specific pathways is unknown in these parasites. Although the kinomes of trypanosomatids are well characterized [37], the signaling pathways may not be necessarily the same as in other organisms like yeasts and mammals. Signaling proteins are expected to regulate gene expression but most of the specific mechanisms and pathways have not been unraveled yet and may be different given the unique features of gene expression in these parasites (reviewed in [38]). The unknown specific function of this protein in signaling may be especially important in *Leishmania* spp. for resistance of amastigotes inside the parasitophorous vacuole of the host phagocyte cell whereas the only colonization step of the *Crithidia* spp. life cycle is the infection of the gut of the insect host. CACK and LACK seem to be important for growth and/or development of the motile stages of the respective species they belong to, as up-regulation in *C. fasciculata* logarithmic phase choanomastigotes has been found herein and its leishmanial orthologue is one of the 50 most abundant transcripts of *L. major* promastigotes [39].

### Changes in abundance of proteins involved in thiol-based redox homeostasis

**Figure 5:**
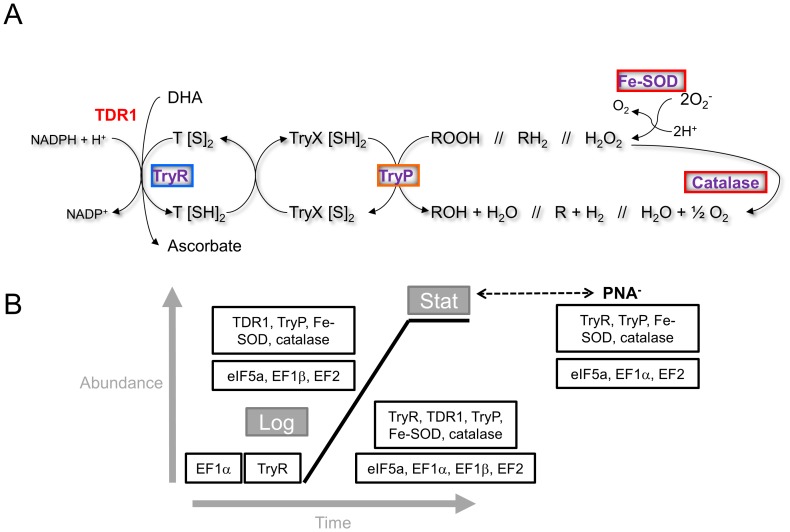
Differentially expressed proteins involved in redox homeostasis and translation in *C. fasciculata* choanomastigotes. (A) The redox control system. Legend: proteins/protein variants in blue are constitutively expressed throughout the growth curve; proteins/protein variants in red are up-regulated at day 1 or 2 (logarithmic phase); proteins in purple are up-regulated in PNA^-^ choanomastigotes. (B) Summary of differential abundance of translation factors and enzymes involved in redox homeostasis throughout the growth curve and in the PNA^-^ subpopulation of choanomastigotes in stationary phase.

The TryP of *C. fasciculata* previously characterized [40] (gi3851500; CfaCl_10_1430) is over-expressed in mid logarithmic phase choanomastigotes, as well as the thiol-dependent reductase 1 (TDR1) (Table 1, Fig. 5). This is an important difference with promastigotes of *L. major*, an ethiological agent of cutaneous leishmaniasis in the Old World, as TryP is constitutively expressed in all the stages of its life cycle [41], [42]. It is also known that *L. donovani* amastigotes up-regulate the TryP with respect to promastigotes [27]. The catalase is absent in pathogenic trypanosomatids [43] but not in *C. fasciculata*. In fact, variants matching with the annotations CfaCl_30_0050 have been identified in several spots (Tables 1 and 3). For this reason, hydrogen peroxide removal in *C. fasciculata* is not necessarily dependent on trypanothione-linked peroxidases, as a difference with the pathogenic trypanosomatids. Like the TryP, this protein is more abundant at mid logarithmic phase. These data suggest higher levels of oxidative stress counteracted with TDR1, TryP and catalase up-regulation in *Crithidia* spp. choanomastigotes than in *Leishmania* spp. promastigotes at mid logarithmic phase, possibly due to the greater growth rate observed in the former (Fig. 1). In fact, the stationary phase is reached about 5–7 days in a typical growth curve of *Leishmania* spp. (e.g., [24]). The up-regulation of TDR1 suggests that the glutathione-ascorbate cycle is also participating in counteracting oxidative stress.

The analysis of the PNA^+^ and PNA^-^ subpopulations within the stationary phase has revealed that the entire redox defense system is up-regulated in the latter (Table 3). This includes the catalase (three variants), the iron superoxide dismutase (Fe-SOD) (two variants), a hypothetical protein (CfaC1_05_0410) orthologue to the trypanothione reductases (TryR) of the pathogenic trypanosomatids and two TryP variants. Therefore, the thiol-based redox defense system is over-expressed in an NADPH-dependent manner in PNA^-^ choanomastigotes. These findings taken together with the expression profile observed throughout the growth curve suggest that the differentiation process of *C. fasciculata* motile choanomastigotes involves an increase in oxidative stress in the axenic culture model that is overcome by the up-regulation of this defense system. These changes have not been observed at the transcript level in *L. infantum* PNA^-^ promastigotes within the stationary phase [11], as well as at the whole stationary phase population at the protein level in *L. infantum* [14] and in *L. major* [41], [42]. The Fe-SOD reduces the superoxide anions generated in the ribonucleotide reductase (RNR) reaction and other reactions to hydrogen peroxide. Then, the catalase and the TryP, catalyze hydrogen peroxide oxidation and peroxidative reactions (reviewed in [44]). The thiol-dependent mechanism also reinforces the reactive oxygen species (ROS) reduction (Fig. 5). This system was characterized in *C. fasciculata* and consists of the enzymes TryR and TryP, also including the tryparedoxin (TryX[S]/[SH_2_]). The trypanothione (T[S]/[SH_2_]) (reviewed in [45]). The T[S]/[SH_2_] consists of two glutathione residues coupled through a spermidine molecule. The peroxiredoxin TryP acts as a catalase and also reducing a variety of other ROS and the TryR regenerates the T[SH_2_] (reduced form) to T[S] (oxidized form).

To summarize, the thiol-based redox control system is over-expressed in mid logarithmic phase *C. fasciculata* choanomastigotes and at the PNA^-^ subpopulation within the stationary phase. The faster growth kinetics compared to *Leishmania* spp. may be related to the higher levels of oxidative stress overcome by the up-regulation of TDR1, TryP and the catalase in *C. fasciculata* rather than in *Leishmania* spp. The up-regulation of the TryR-TryP system together with the catalase and the Fe-SOD in PNA^-^ choanomastigotes suggests a relationship between differentiation and the capability to overcome increased levels of oxidative stress.

### Differences in the proteome profiles of the motile stages of *C. fasciculata* and *Leishmania* spp

Stage-specific regulation at the transcript and protein levels in the genus *Leishmania* has been widely studied. Much of the information extracted is related to differentially regulated genes between amastigotes and promastigotes [27], [29], [41], [46], [47] but few analyses have provided data about differential expression throughout the promastigote growth curve [48], [49], [50]. A high-throughput transcriptome analysis was specifically focused on the differences between logarithmic and stationary phase promastigotes with amastigotes in *L. infantum* [24]. Comparing the proteomic data sets available for the motile stages of *L. infantum* [14] and *C. fasciculata* (this work), an important difference has been found, the up-regulation of the TryR in logarithmic phase promastigotes in the former (this gene was reported as thiol-dependent antioxidant protein, gi21307665 in the NCBI protein databank and corresponds to gene LinJ.05.0350) *vs.* the PNA^-^ subpopulation in *C. fasciculata*.

Important differences with *Leishmania* spp. can be noticed taking the information extracted from the transcriptome and the proteome as a whole. Indeed, regarding metabolic processes, the expression profiles of glucolytic enzymes and the thiolase between the motile stages of *L. infantum* and *C. fasciculata* are different. Glucolysis is active in *Leishmania* spp. promastigotes and probably reaches its highest activity in stationary phase, whereas expression of many of the glucolytic enzymes decays in stationary phase choanomastigotes in *C. fasciculata*, being increased only in PNA^-^ parasites.

As a difference with the *L. major* TryP [40], the *C. fasciculata* orthologue does not maintain constant expression levels in the motile stage, especially in the PNA^-^ subpopulation of choanomastigotes in stationary phase, where the whole thiol-based redox defense system (catalase, Fe-SOD, TryP, TryR) is up-regulated. In fact, differential expression of the TryR, the TryP and the Fe-SOD was not found in *L. major* and *L. infantum* metacyclic promastigotes, whereas the tryparedoxin levels vary between procyclic and metacyclic promastigotes in both species [11], [48], [49]. Additionally, up-regulation of the TryR observed in *L. infantum* logarithmic phase promastigotes has been precisely the only one not found in *C. fasciculata* choanomastigotes, where TDR1 is more abundant instead. The oxidative phase of the pentose-phosphate shunt is the source of NADPH for these important cellular processes and it is constitutively expressed in both organisms probably. However, the non-oxidative phase of this pathway is over-expressed in logarithmic phase only in *C. fasciculata*. The plethora of changes in gene expression regulation and post-translational modification of proteins observed previously in *L. infantum* promastigotes has not been observed in *C. fasciculata* choanomastigotes. Indeed, only the eIF5a, the EF1β, the EF2 and the hsp60 are up-regulated in logarithmic phase choanomastigotes (Table 1) whereas the PABP, two RNAbp, two variants of the EF1α, an ATP-dependent RNA helicase, a different hsp60 variant, the Nkdb, a DnaJ chaperone and a peptidyl-prolyl cis-trans isomerase and two cyclophilins are constitutively expressed throughout the growth curve (Table 2) according to this analysis. A different profile of EF2 expression was observed in *L. major*, where it is over-expressed in metacyclic promastigotes [48]. Interestingly, the EF1α is more abundant in PNA^-^ choanomastigotes of *C. fasciculata*, what is opposite to PNA^-^ promastigotes of *L. infantum*, where it decreases. This may be linked with differences between the developmental processes these parasites undergo instead of being related to growth in a rich medium and contributes to explain in part the differences between the life cycles of *C. fasciculata* and *Leishmania* spp. Promastigotes undergo a deep differentiation process inside the gut of the phlebotomine sand fly to successfully establish intracellular infection in the mammalian host. By contrast, the life cycle of *Crithidia* spp. is monogenetic and differentiation of the characteristic choanomastigote motile stage is probably not so complex because the amastigote stage is not intracellular. This assumption is suggested by the substantial differences found in the abundance of proteins involved in gene expression regulation and protein modification between both genera. However, both organisms are close in the crown of the phylogenetic tree of trypanosomatids and growth kinetics of their motile stages in culture is similar. For this reason, future in depth study of these differences in protein abundance may help to explain their different mechanisms of adaptation to axenic cultures that mimic the conditions of the insect host gut. The MASCOT identifications performed against the *NCBInr* database (Tables S1-S3 in S1 File) provide additional evidence for the hypothesis of a very close relationship in the evolutionary tree. The increased abundance of the translation factors eIF5a, EF1β the EF2 in early logarithmic phase choanomastigotes may be linked to a more active metabolic status required for growth, whereas the increase in eIF5a, EF1α and EF2 in the PNA^-^ choanomastigote subpopulation in stationary phase suggests a role in development, as it cannot be associated to growth under starvation conditions (Fig. 5B). The same has been observed with the redox homeostasis control system. Logarithmic phase *C. fasciculata* choanomastigotes up-regulate the ascorbate-dependent TDR1, as well as the TryP, the catalase and the Fe-SOD, probably because of an increased oxidative stress resulting from faster growth kinetics than in *Leishmania* spp., and the abundance of these enzymes decay in the ongoing of the growth curve. Then, it increases again in the PNA^-^ subpopulation within the stationary phase (TryR instead of TDR1), being presumably associated to development at this stage as well (Fig. 5B). It is important to notice that the expression profiles described correspond to the motile stages of these parasites in axenic culture and the developmental processes of *Crithidia* spp. are almost unexplored. The axenic culture model is widely accepted in *Leishmania* spp. because cultured promastigotes are able to stablish infection in mammalian hosts, what suggests that the main developmental processes may be reproduced in culture. In the case of *Crithidia* spp., it is not known whether culture greatly affects development. The main findings of this study point to similar but faster growth kinetics in *C. fasciculata* and different metabolic adaptations to the same culture medium conditions.

## Conclusions

The growth kinetics is slightly faster in *C. fasciculata* than in *Leishmania* spp. Choanomastigotes of *C. fasciculata* are able to agglutinate with PNA and a non-agglutinating subpopulation can be isolated. Consequently, the behavior in the presence of the lectin is the same. Logarithmic phase choanomastigotes of *C. fasciculata* over-express CACK, enzymes involved in redox homeostasis (TDR1, TryP, catalase and Fe-SOD), the translation factors eIF5a, EF1β and EF2 and most of the glycolytic enzymes catalyzing irreversible reactions and the enzymes of the non-oxidative phase of the pentose-phosphate pathway. The abundance of the translation factors (EF1α instead of EF1β) and of the enzymes involved in redox homeostasis (TryR instead of TDR1) increases again in the PNA^-^ subpopulation. These changes in abundance may have a role in growth in the nutrient rich environment at the logarithmic phase and a role in differentiation in the minor PNA^-^ subpopulation within the population in stationary phase.

## Supporting Information

S1 File **Supporting tables.** Table S1. Differentially regulated proteins throughout the growth curve of *C. fasciculata* choanomastigotes identified with the *NCBInr* database. Estimated pI, significant MASCOT scores and ratios to day 1 are provided. Only spots with statistically significant ratios (p<0.05) over 1.7 or under 0.6 were picked and analyzed and are shown in the table. As a consequence, hyphens in the columns containing ratios do not necessarily indicate lack of differential abundance, because there are also cases of lack of statistical significance of ratios indicating over- or under-expression. Table S2. Constantly expressed proteins throughout the growth curve of *C. fasciculata* choanomastigotes identified with the *NCBInr* database. Estimated molecular weights, pI and significant MASCOT scores are provided. Table S3. Differential abundance of identified proteins between the PNA^+^ and PNA^-^ subpopulations of *C. fasciculata* choanomastigotes in stationary phase of axenic culture identified with the *NCBInr* database. Estimated molecular weights, pI, significant MASCOT scores and PNA^+^/PNA^-^ ratios. Only spots with statistically significant ratios (p<0.05) over 1.7 or under 0.6 were picked and analyzed and are shown in the table.
(DOCX)

S2 File **Supporting figures.** Figure S1. Western blot of *C. fasciculata* choanomastigote protein extracts throughout the growth curve for CACK detection. Complete image of the autoradiography. Figure S2. Western blot of *C. fasciculata* choanomastigote protein extracts throughout the growth curve for gGAPDH detection. Complete image of the autoradiography.
(PPT)
