# Supplementary material for: An Insight into the Proteome of Crithidia fasciculata Choanomastigotes as a Comparative Approach to Axenic Growth, Peanut Lectin Agglutination and Differentiation of Leishmania spp. Promastigotes
Source: PLoS One. 2014 Dec 11;9(12):e113837. doi: 10.1371/journal.pone.0113837 (PMC4263474; doi:10.1371/journal.pone.0113837)
Supplement: S1 File — Supporting tables. Table S1. Differentially regulated proteins throughout the growth curve of C. fasciculata choanomastigotes identified with the NCBInr database. Estimated pI, significant MASCOT scores and ratios to day 1 are provided. Only spots with statistically significant ratios (p<0.05) over 1.7 or under 0.6 were picked and analyzed and are shown in the table. As a consequence, hyphens in the columns containing ratios do not necessarily indicate lack of differential abundance, because there are also cases of lack of statistical significance of ratios indicating over- or under-expression. Table S2. Constantly expressed proteins throughout the growth curve of C. fasciculata choanomastigotes identified with the NCBInr database. Estimated molecular weights, pI and significant MASCOT scores are provided. Table S3. Differential abundance of identified proteins between the PNA+ and PNA- subpopulations of C. fasciculata choanomastigotes in stationary phase of axenic culture identified with the NCBInr database. Estimated molecular weights, pI, significant MASCOT scores and PNA+/PNA- ratios. Only spots with statistically significant ratios (p<0.05) over 1.7 or under 0.6 were picked and analyzed and are shown in the table. (DOCX) [file pone.0113837.s001.docx]

**Table S1. Differentially regulated proteins throughout the growth curve of *C. fasciculata* choanomastigotes identified with the *NCBInr* database.** Estimated pI, significant MASCOT scores and ratios to day 1 are provided. Only spots with statistically significant ratios (p < 0.05) over 1.7 or under 0.6 were picked and analyzed and are shown in the table. As a consequence, hyphens in the columns containing ratios do not necessarily indicate lack of differential abundance, because there are also cases of lack of statistical significance of ratios indicating over- or under-expression.

| **Spot** | **Protein (species)** | **NCBI Acc. no.** (gi) | **MW** (KDa) | **pI** | ***MASCOT* score**  (p<0.05) | **Ratio** (p < 0.05) | | |
| --- | --- | --- | --- | --- | --- | --- | --- | --- |
|  |  |  |  |  |  | d2:d1 | d3:d1 | d4:d1 |
| Cf1101 | β-tubulin (*L. braziliensis*) | 154343842 | 17.9 | 4.2 | 73 | 0.01 | _ | _ |
| Cf1102 | β-tubulin (*L. braziliensis*) | 154343842 | 22.2 | 4.4 | 91 | 0.58 | _ | _ |
| Cf1103 | Putative eukaryotic initiation factor 5a (*L. infantum*)/β-tubulin (*L. braziliensis*) | 146088631/ 154343842 | 23.6 | 4.6 | 177/80 | 0.48 | _ | _ |
| Cf1202 | β-tubulin (*L. donovani*) | 262233287 | 26.8 | 4.4 | 64 | 0.44 | _ | _ |
| Cf1801 | Peroxisomal targeting signal-1 receptor (*L. donovani*) | 7715049 | 92.6 | 4.3 | 78 | _ | 0.32 | _ |
| Cf1802 | Hypothetical protein (*L. braziliensis*) | 154335998 | 70.4 | 4.4 | 56 | _ | _ | 1.72 |
| Cf2303 | β-tubulin (*L. donovani*) | 262233287 | 33.6 | 4.9 | 408 | 0.24 | _ | _ |
| Cf2401 | α-tubulin (*L. braziliensis*)/β-tubulin (*L. mexicana*) | 322504442/ 322488903 | 35.0 | 5.2 | 399/152 | 0.21 | _ | _ |
| Cf2903 | α-tubulin (*L. braziliensis*) | 322504442 | 227.4 | 4.7 | 300 | 89.60 | 0.18 | _ |
| Cf3203 | Tryparedoxin peroxidase (*C. fasciculata*) | 3851500 | 24.1 | 5.4 | 246 | _ | 3.28 | _ |
| Cf3401 | Transaldolase B (*Bordetella parapertussis*) | 33595900 | 36.1 | 5.6 | 110 | 0.46 | _ | _ |
| Cf3403 | CACK protein (*C. fasciculata*) | 3132790 | 36.5 | 5.7 | 211 | 0.58 | _ | _ |
| Cf3605 | Actin (*L. mexicana*) | 322488108 | 46.9 | 5.6 | 109 | 2.34 | _ | _ |
| Cf3608 | Enolase (*L. mexicana*) | 322489720 | 51.1 | 5.4 | 186 | 1.73 | _ | _ |
| Cf3701 | Chaperonin hsp60, mitochondrial precursor (*L. major*) | 157876872 | 67.5 | 5.5 | 245 | _ | 0.01 | _ |
| Cf3703 | Vacuolar ATP synthase subunit B (*L. braziliensis*) | 154340667 | 55.8 | 5.5 | 236 | 8.41 | _ | _ |
| Cf4303 | Putative GTP-binding protein (*L. infantum*) | 146088901 | 29.1 | 5.8 | 97 | _ | _ | 1.76 |
| Cf4501 | Enolase (*L. donovani*) | 190335775 | 41.0 | 5.7 | 159 | 0.50 | _ | _ |
| Cf4503 | Enolase (*L. infantum*) | 146081643 | 41.0 | 5.8 | 190 | 0.56 | _ | _ |
| Cf4602 | Putative glutamate dehydrogenase (*L. mexicana*) | 322493379 | 50.2 | 5.7 | 210 | 2.12 | _ | _ |
| Cf5001 | Tryparedoxin peroxidase (*C. fasciculata*) | 3851500 | 12.9 | 5.8 | 258 | 0.02 | _ | _ |
| Cf5301 | Putative glycosomal malate dehydrogenase (*T. congolense*) | 342185054 | 34.2 | 5.9 | 113 | 2.15 | _ | 2.85 |
| Cf5502 | Alcohol dehydrogenase (*T. cruzi*) | 71660317 | 40.87 | 6.0 | 77 | _ | 0.58 | _ |
| Cf5601 | Enolase (*L. infantum*) | 146081643 | 51.2 | 6.0 | 478 | 1.84 | _ | _ |
| Cf5701 | Conserved hypothetical protein (*L. mexicana*) | 322492269 | 55.9 | 5.9 | 145 | 0.58 | _ | _ |
| Cf5703 | Pyruvate kinase (*L. braziliensis*) | 154344357 | 59.3 | 6.1 | 132 | 3.94 | _ | _ |
| Cf6002 | Calpain-like cysteine peptidase, ClanCA, family C2 (*L. infantum*) | 157868661 | 15.4 | 6.2 | 75 | 0.50 | 1.70 | _ |
| Cf6003 | Unnamed protein product (*L. donovani*) | 322503374 | 12.9 | 6.1 | 158 | 0.01 | _ | _ |
| Cf6101 | Tryparedoxin peroxidase (*L. major*)/Putative iron superoxide dismutase (*T. vivax*) | 160347104/ 340059792 | 21.8 | 6.2 | 96/79 | 0.58 | 1.72 | _ |
| Cf6503 | NADP-dependent alcohol dehydrogenase (*L. braziliensis*) | 154337772 | 40.4 | 6.2 | 108 | 1.93 | _ | _ |
| Cf6603 | Enolase (*L. infantum*) | 146081643 | 51.2 | 6.0 | 164 | 80.53 | _ | _ |
| Cf7302 | Putative fructose-1,6-bisphosphate aldolase (*Crithidia* sp.) | 242133533 | 32.9 | 6.6 | 145 | 0.23 | _ | _ |
| Cf7402 | Putative fructose-1,6-bisphosphate aldolase (Crithidia sp.) | 242133533 | 36.8 | 6.6 | 155 | 0.35 | 3.02 | _ |
| Cf7501 | Putative phosphoribosyl transferase (*L. infantum*) | 146097960 | 42.7 | 6.5 | 45 | 2.6 | _ | _ |
| Cf7603 | Putative 3-ketoacyl-CoA thiolase, fragment (*T. vivax*) | 340055279 | 47.4 | 7.3 | 119 | 1.74 | _ | _ |
| Cf7703 | Succinyl-CoA:3-ketoacid-coenzyme A transferase, mitochondrial precursor (*L. braziliensis*) | 154344174 | 56.7 | 6.7 | 109 | 2.22 | _ | 2.14 |
| Cf7704 | Putative dihydrolipoamide dehydrogenase (*L. infantum*) | 146097055 | 54.8 | 6.8 | 212/90 | 2.72 | _ | _ |
| Cf8402 | Malate dehydrogenase (*L. infantum*) | 146098508 | 34.9 | 7.6 | 188 | 2.38 | _ | _ |
| Cf8504 | Putative fructose-1,6-bisphosphate aldolase (*Crithidia* sp.) | 242133533 | 42.8 | 8.7 | 167 | 2.93 | _ | _ |
| Cf8601 | Hexokinase (*L. major*) | 157868810 | 49.3 | 8.8 | 224 | 4.62 | _ | 4.83 |
| Cf8701 | Hypothetical protein (*L. braziliensis*) | 154343009 | 62.5 | 8.0 | 112 | 0.50 | _ | _ |
| Cf9302 | Putative glycosomal malate dehydrogenase (*T. congolense*) | 342185054 | 33.7 | 9.3 | 192/65 | 1.99 | _ | _ |

**Table S2. Constantly expressed identified proteins throughout the growth curve of *C. fasciculata* choanomastigotes identified with the *NCBInr* database.** Estimated molecular weights, pI and significant MASCOT scores are provided.

| **Spot** | **Protein (species)** | **NCBI Acc. no.** (gi) | **MW** (KDa) | **pI** | **MASCOT score** (p < 0.05) |
| --- | --- | --- | --- | --- | --- |
| Cf1001 | Calpain-like cysteine peptidase (*L. braziliensis*) | 154334241 | 17.91 | 4.5 | 282 |
| Cf1602 | Hypothetical protein conserved (*L. major*) | 157867582 | 52.82 | 4.6 | 76 |
| Cf1904 | Hypothetical protein (*L. braziliensis*) | 154337505 | 105.02 | 4.4 | 103 |
| Cf2702 | β-tubulin (*L. Infantum*) | 322488903 | 60.31 | 4.7 | 490 |
| Cf2703 | α-tubulin (*L. infantum*) | 322504442 | 57.83 | 5.0 | 386 |
| Cf2805 | Heat shock protein 70 | \| [239580147](http://procyon/mascot/cgi/master_results.pl?REPTYPE=Protein&file=E:/MASCOT/data/20120615/F088326.dat#Hit1) \|  \| \| --- \| --- \| | 76.77 | 5.3 | 744 |
| Cf2901 | Putative calpain-like cysteine peptidase (*L. braziliensis*) | 322504829 | 171.73 | 4.7 | 87 |
| Cf4201 | α- tubulin (*L. braziliensis*) | 154333816 | 25.11 | 5.7 | 62 |
| Cf4401 | Unnamed protein product (*L. mexicana*) | 322488761 | 36.76 | 5.8 | 72 |
| Cf6401 | Coproporphyrinogen III oxidase (*Endotrypanum costaricensis*) | 343433415/343433419 | 35.33 | 6.1 | 154/103 |
| Cf6501 | NADP-dependent alcohol dehydrogenase (*L. braziliensis*) | 154337772 | 40.91 | 6.0 | 82 |
| Cf6901 | Aconitase (*L. major*) | 157867807 | 104.44 | 6.4 | 128 |
| Cf7002 | Nucleoside diphosphate kinase (*T. brucei*) | 74026208 | 16.09 | 6.7 | 187 |
| Cf7204 | Hypothetical protein | 326436349 | 27.71 | 7.5 | 72 |
| Cf7301 | RNA-binding protein (*L. braziliensis*) | 154344761 | 37.67 | 6.5 | 201 |
| Cf7602 | Phosphoglycerate kinase, cytosolic | 129909 | 46.54 | 6.5 | 638 |
| Cf8102 | Peptidyl-prolyl cis-trans isomerasa (*Ogataea parapolymorpha*) | 320583666 | 19.39 | 8.6 | 94 |
| Cf8104 | Cyclophilin (*L. infantum*) | 71412806 | 22.57 | 9.1 | 159 |
| Cf8202 | Triose phosphate isomerase (*L. braziliensis*) | 322504827 | 24.89 | 7.5 | 128 |
| Cf8203 | Triose phosphate isomerase (*L. braziliensis*) | 322504827 | 25.02 | 8.3 | 136 |
| Cf8302 | Glycosomal malate dehydrogenase (*T. brucei*) | 71749346 | 33.63 | 8.7 | 88 |
| Cf8702 | Poly(A)-binding protein (*C. fasciculata*) | 52221255 | 68.60 | 9.0 | 219 |
| Cf9301 | Glycosomal malate dehydrogenase (*T. brucei*) | 71749346 | 31.77 | 9.2 | 101 |
| Cf9401 | Elongation factor 1α, putative (*Leptomonas podlipaevi*) | 226347441 | 36.35 | 9.3 | 155 |
| Cf9601 | Elongation factor 1α, partial | 226347441 | 53.84 | 9.2 | 165 |

**Table S3. Differential abundance of identified proteins between the PNA^+^ and PNA^-^ subpopulations *C. fasciculata* choanomastigotes in stationary phase of axenic culture identified with the *NCBInr* database.** Estimated molecular weights, pI, significant MASCOT scores and PNA^+^/PNA^-^ ratios. Only spots with statistically significant ratios (p < 0.05) over 1.7 or under 0.6 were picked and analyzed and are shown in the table.

| **Spot** | **Protein** | **NCBI Acc. no.**  gi\| | **MW** (KDa) | **pI** | ***MASCOT* score** (p < 0.05) | **Ratio** (p < 0.05)  (PNA^+^):(PNA^-^) |
| --- | --- | --- | --- | --- | --- | --- |
| Cf0104 | β-tubulin (*L. braziliensis*)/Eukaryotic initiation factor 5a (*L. infantum*) | 154343852/146088631 | 20.52 | 3.5 | 172/68 | 0.44 |
| Cf_p_1203 | β-tubulin (*L. donovani*) | 262233287 | 35.48 | 3.8 | 395 | 0.17 |
| Cf_p_1803 | Heat shock protein 70 (L. braziliensis) | 9864199 | 86.91 | 4.7 | 716 | 0.41 |
| Cf_p_1901 | Conserved hypothetical protein (*L. mexicana*) | 322491767 | 142.13 | 4.3 | 112 | 0.14 |
| Cf_p_2801 | 2,3-bisphosphoglycerate-independent phosphoglycerate mutase, putative (*T. cruzi*) | 322820998 | 76.42 | 5.0 | 71 | 0.40 |
| Cf_p_2802 | Heat shock protein 70, mitochondrial precursor (*L. major*) | 157872646 | 88.35 | 5.1 | 204 | 0.15 |
| Cf_p_3101 | Tryparedoxin peroxidase (*C. fasciculata*) | 3851500 | 22.36 | 5.4 | 254 | 0.01 |
| Cf_p_4002 | α-tubulin (*L. donovani*) | 51847763 | 10.52 | 5.5 | 114 | 0.03 |
| Cf_p_4202 | GTP-binding protein (*L. infantum*) | 146088901 | 23.10 | 5.6 | 98 | 0.03 |
| Cf_p_5001 | Tryparedoxin peroxidase (*L. major*) | 160347104 | 14.23 | 5.9 | 61 | 0.56 |
| Cf_p_5101 | Tryparedoxin peroxidase (*C. fasciculata*) | 3851500 | 20.46 | 5.7 | 281 | 0.46 |
| Cf_p_5301 | Enolase (*L. mexicana*)/Enolase (*T. brucei*) | 322489720/556208 | 41.26 | 5.7 | 246/82 | 0.33 |
| Cf_p_5701 | Chain A, substrate interactions between trypanothione reductase | 494695 | 60.28 | 5.7 | 117 | 0.31 |
| Cf_p_5702 | Conserved hypothetical protein (*L. mexicana*) | 322492269 | 47.91 | 5.9 | 99 | 0.57 |
| Cf_p_5901 | Elongation factor 2 (*L. braziliensis*) | 154345432 | 171.5 | 5.8 | 49 | 0.54 |
| Cf_p_6001 | Chain A, cristal structure of the enzyme Fe-superoxide dismutase Tbsodb2 (*T.brucei*) | 237640521 | 19.25 | 6.0 | 68 | 0.59 |
| Cf_p_6401 | Coproporphyrinogen III oxidase (*Azoarcus* sp.) | 119899186 | 38.52 | 6.1 | 103 | 0.28 |
| Cf_p_7502 | GTP-binding protein (*L. infantum*) | 146088901 | 53.96 | 6.3 | 98 | 0.64 |
| Cf_p_7702 | Catalase (*Nitrosomonas* sp.) | 325983250 | 87.42 | 6.2 | 114 | 0.53 |
| Cf_p_7704 | Catalase (*Nitrosomonas* sp.) | 325983250 | 87.11 | 6.3 | 123 | 0.36 |
| Cf_p_7804 | Fumarate reductase (*L. tropica*) | 94469897 | 98.56 | 6.1 | 71 | 0.06 |
| Cf_p_9602 | Elongation factor 1 (*Pseudomonas podlipaevi*) | 226347441 | 71.43 | 9.3 | 380 | 0.01 |
| Cf_p_1301 | α-tubulin (*L. braziliensis*) | 322504442 | 36.09 | 4.6 | 143 | 3.31 |
| Cf_p_7302 | Putative fructose-1,6-bishosphate aldolase (*Crithidia* sp.) | 242133533 | 39.05 | 6.2 | 73 | 1.92 |
| Cf_p_8701 | Putative succinyl-CoA: 3-ketoacid-CoA transferase, mitochondrial precursor (*L. mexicana*) | 322494738 | 75.28 | 6.5 | 83 | 2.01 |
| Cf_p_9203 | Glycosomal malate dehydrogenase (*T. brucei*) | 71749346 | 35.74 | 9.6 | 123 | 3.16 |
| Cf_p_9501 | Putative fructose-1,6-bisphosphate aldolase (*Crithidia* sp.)/Predicted protein (*Phaeodactylon tricornutum*) | 242133533/219111629 | 48.93 | 9.2 | 96/76 | 1.89 |
| Cf_p_9601 | Hexokinase (*L. major*) | 157868810 | 61.24 | 9.2 | 206 | 7.26 |
