# Supplementary material for: An Insight into the Proteome of Crithidia fasciculata Choanomastigotes as a Comparative Approach to Axenic Growth, Peanut Lectin Agglutination and Differentiation of Leishmania spp. Promastigotes
Source: PLoS One. 2014 Dec 11;9(12):e113837. doi: 10.1371/journal.pone.0113837 (PMC4263474; doi:10.1371/journal.pone.0113837)
Supplement: S2 File — Supporting figures. Figure S1. Western blot of C. fasciculata choanomastigote protein extracts throughout the growth curve for CACK detection. Complete image of the autoradiography. Figure S2. Western blot of C. fasciculata choanomastigote protein extracts throughout the growth curve for gGAPDH detection. Complete image of the autoradiography. (PPT) [file pone.0113837.s002.ppt]

## Slide 1
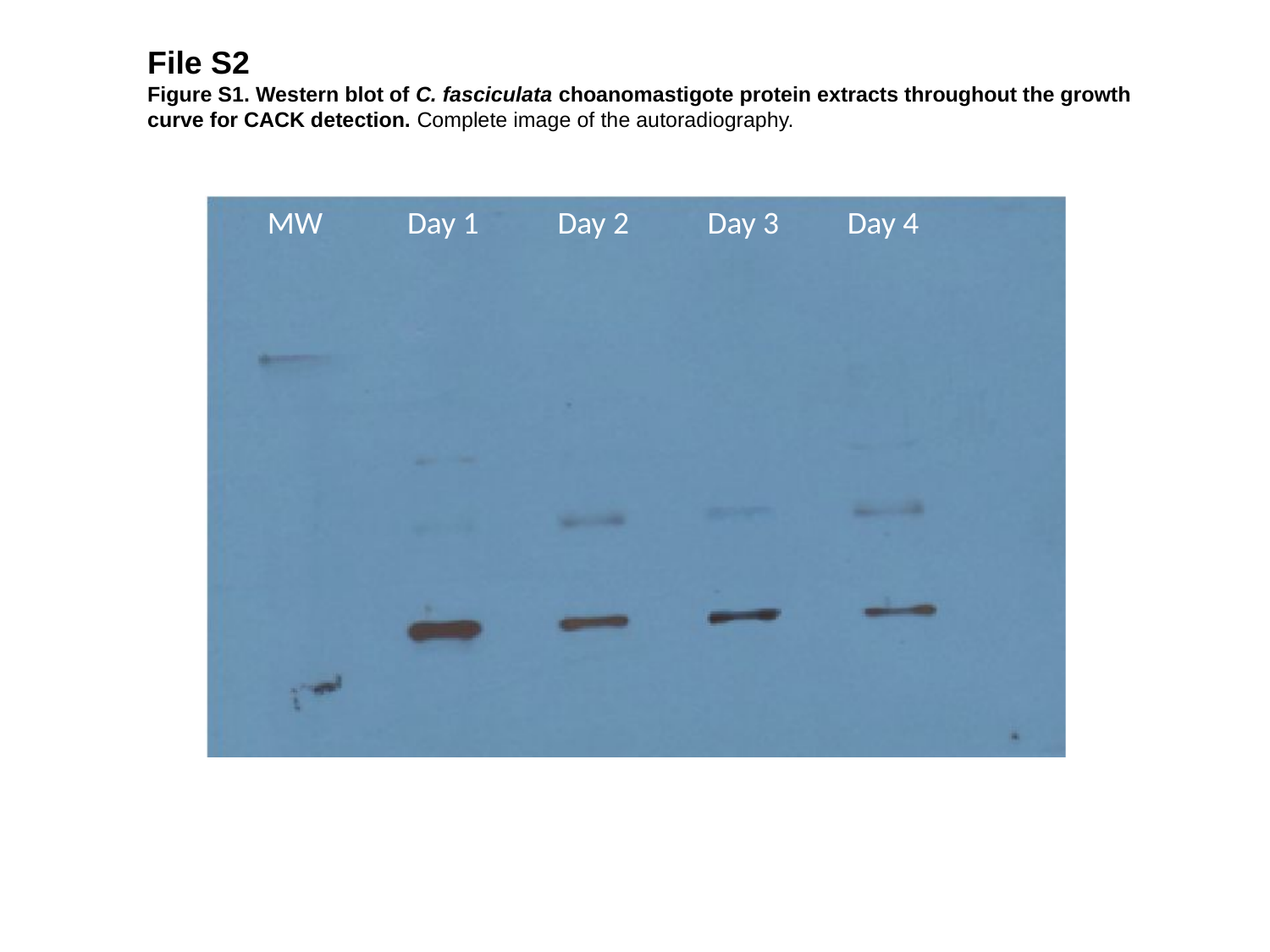

File S2
Figure S1. Western blot of C. fasciculata choanomastigote protein extracts throughout the growth curve for CACK detection. Complete image of the autoradiography.
MW
Day 1
Day 2
Day 3
Day 4

## Slide 2
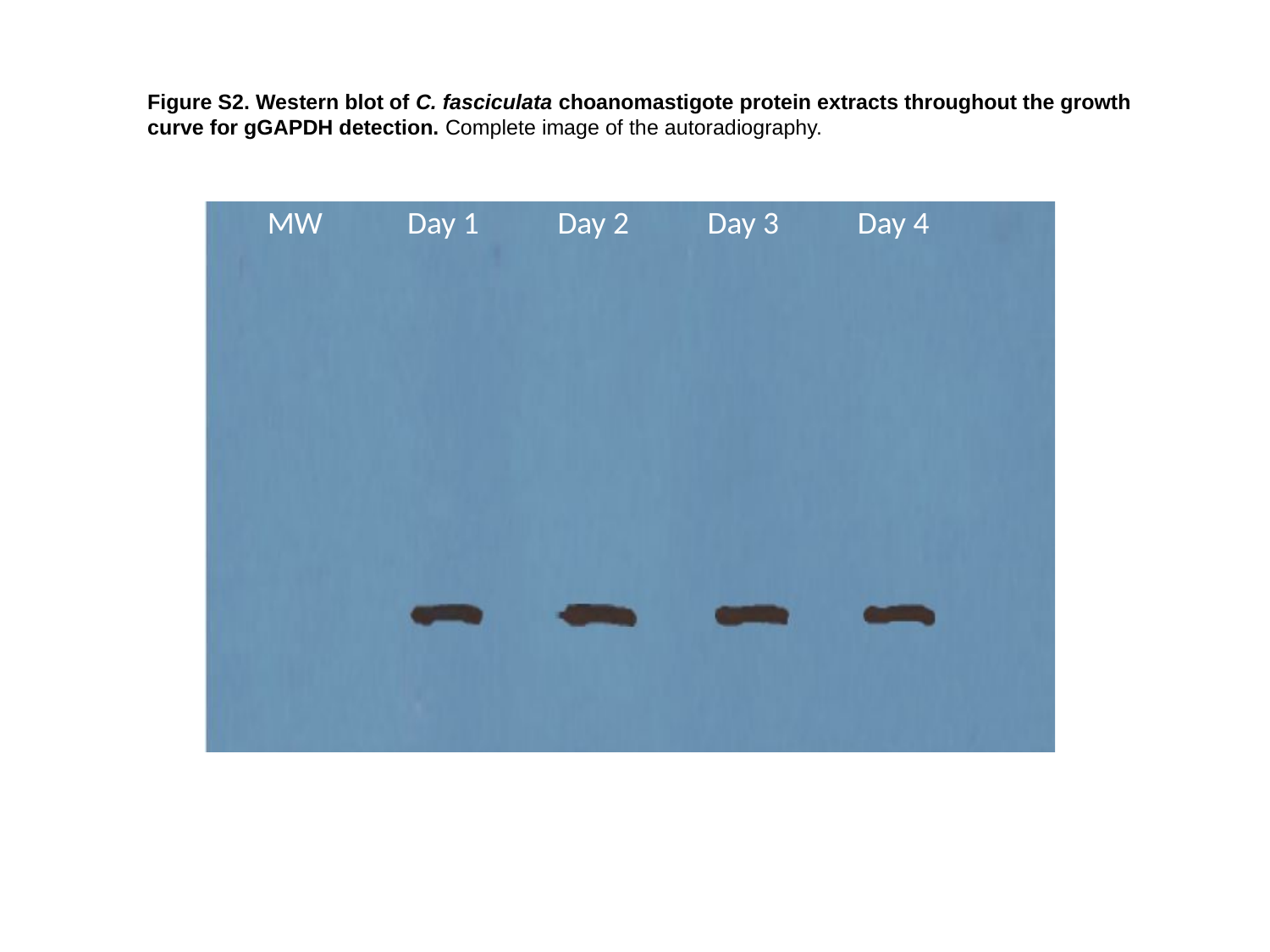

Figure S2. Western blot of C. fasciculata choanomastigote protein extracts throughout the growth curve for gGAPDH detection. Complete image of the autoradiography.
MW
Day 1
Day 2
Day 3
Day 4
